# Variation in health warning effectiveness on cigarette packs: a need for regulation?

**DOI:** 10.1093/eurpub/ckw094

**Published:** 2016-07-06

**Authors:** Olivia M. Maynard, Monika Misak, Marcus R. Munafò

**Affiliations:** 1 School of Experimental Psychology, University of Bristol, Bristol, UK; 2 MRC Integrative Epidemiology Unit (IEU), University of Bristol, Bristol, UK; 3 UK Centre for Tobacco and Alcohol Studies, UK; 4 The City College of New York, The Sophie Davis School of Biomedical Education, New York, NY, USA

## Abstract

The Tobacco Products Directive allows the possibility of strategic placement of health warnings on cigarette packs by manufacturers to reduce overall warning effectiveness. Information regarding health warning effectiveness was assessed in an online survey, and the prevalence of warnings on cigarette packs was assessed in a shop survey. Although we find no evidence of a strong correlation between health warning effectiveness ratings and their frequency on cigarette packs (*r* = −0.17, *P* = 0.56), there may be other ways this possibility is exploited. We suggest that this potential loophole is addressed and monitoring of the placement of health warnings on cigarette packs is continued.

## Introduction

Cigarette pack health warnings are designed to increase health knowledge and perceptions of risk of smoking and can promote smoking cessation.^1^ The 14 pictorial warnings placed on the back of cigarette packages in the UK between October 2008 and May 2016 were taken from a larger set of warnings specified by the European Commission Tobacco Products Directive (TPD; 2001/37/EC). Article 10 of the TPD states that warnings ‘shall be rotated in such a way as to guarantee their regular appearance’, although ‘regular’ is not defined. This offers an opportunity for tobacco companies to strategically place health warnings on cigarette packs in order to reduce their overall effectiveness.

Strategic placement could be achieved in a number of ways. First, given warnings vary in their effectiveness,^1^^,^^2^ less effective warnings could more frequently be placed on cigarette packs than more effective ones. Second, warnings designed for certain target populations could be placed on packs which these populations less frequently purchase (i.e. warnings that specifically address the negative impacts of smoking for men could be placed more frequently on packs more popular among women).^3^ While the updated TPD (2014/40/EU), which came into force in May 2016, provides more specific guidance on the placement of health warnings (i.e. ‘the producer … must select the graphic … so that each of the 14 graphics in that set appears on between 1/24 and 1/12 of the total number of packs …’), these regulations still allow tobacco companies to exploit this loophole.

The primary aim of this study was to examine adherence to this TPD requirement for the first time and explore whether there is variation in the prevalence of certain warnings on cigarette packs. Specifically, we aimed to address the following questions: (i) Are less effective health warnings found more frequently on cigarette packs than more effective health warnings? (ii) Are female-specific health warnings found less frequently on female-specific cigarette packs than male-specific or gender-neutral health warnings? Information regarding health-warning effectiveness and gender specificity of warnings and cigarette brands was assessed in an online survey and the prevalence of health warnings on packs was assessed in a shop survey.

## Methods

The protocol for both the online survey and the shop survey was published online on the Open Science Framework prior to testing (https://osf.io/vft3k/). The data that form the basis of the results presented here are available from the Bristol Research Data Repository (http://data.bris.ac.uk/data; doi: 10.5523/bris.1sx8k1da7jfe615qyaazn3hvh0). Ethics approval was obtained from the Faculty of Science Research Ethics Committee at the University of Bristol (ethics approval code: 24761).

### Online survey

Participants in the online survey provided demographic information, including their smoking status, and were then shown each of the 14 pictorial health warnings^4^ used in the UK (prior to May 2016) in turn. Participants responded to two questions: (i) Whether the warning is ‘more aimed at females’, ‘more aimed at males’ or ‘aimed at both males and females’ and (ii) ‘Overall, on a scale from 1 to 10, how effective is this health warning?’.^5^ Participants were then shown 16 cigarette packs in turn and asked to respond to a single question: whether the pack is ‘more aimed at females’, ‘more aimed at males’ or ‘aimed at both males and females’.

### Shop survey

Between July and August 2015, researchers visited shops selling tobacco in Bristol, UK, and went behind the counter to record the health warnings placed on the back of each of the 16 cigarette packs assessed in the online survey. Our sample size calculation estimated that we required 1000 cigarette packs to investigate the relationship between warning effectiveness and frequency. Further details are provided in the study protocol. A further 1000 female-specific cigarette packs were required to investigate the relationship between cigarette pack gender specificity and warning frequency.

## Results

### Online survey

**Figure 1:**
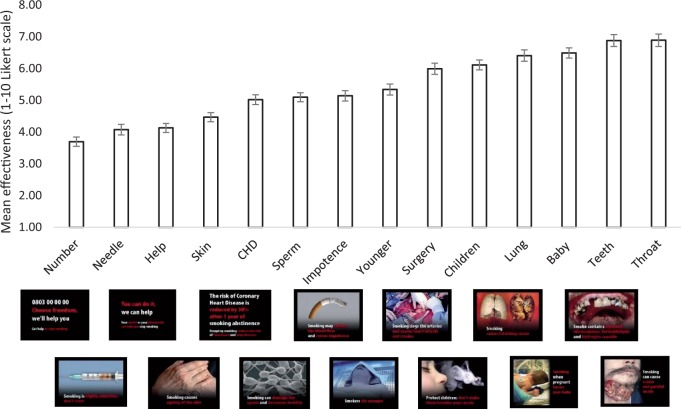
Mean effectiveness scores for each of the health warnings among all participants. Error bars represent standard error of the mean

A total of 206 participants (48.8% female) provided data, although five participants were removed from data analysis as demographic data were not collected. Participants had a mean age of 30.7 years (SD 13.5). Seventy-five percent of participants were non-smokers or ex-smokers. There was wide variation in the perceived effectiveness scores for the warnings (figure 1). An independent samples *t*-test indicated no evidence (*t*_(110.7)_ = 0.77, *P* = 0.44) for a difference in perceived effectiveness ratings between non-smokers (mean 5.45, SD 1.72) and smokers (mean 5.28, SD 1.30). Further analyses were combined across smoking groups.

Four cigarette packs (all of the ‘Vogue’ brands) were identified as being female specific (i.e. at least 70% of participants reported that these packs were aimed at females). No packs were identified as being male specific. Only one health warning was identified as being female specific using this same criterion (‘Smoking when pregnant harms your baby’). Two warnings were identified as being male-specific (‘Smoking may reduce the blood flow and causes impotence’ and ‘Smoking can damage the sperm and decreases fertility’).

### Shop survey

The health warnings on a total of 1,440 cigarette packs were recorded. If the distribution of the 14 warnings was equal across packs, we would have expected to observe 103 of each warning. However, we observed considerable variation in health warning prevalence. The least common warning was the ‘CHD’ warning (figure 1), appearing on 78 packs. The most common warning was the ‘surgery’ warning, appearing on 130 packs. However, there was no evidence of a strong correlation between warning effectiveness ratings and their frequency (*r* = −0.17, *P* = 0.56).

Only 29 female-specific packs were identified: 17 Vogue Bronze (10 ‘surgery’ warnings, 7 ‘help’ warnings), 9 Vogue Menthe (6 ‘younger’ warnings, 3 ‘surgery’ warnings), 3 Vogue Menthe Superslim (3 ‘number’ warnings) and 0 Vogue Bleue Superslim. Given the small sample size, statistical tests were not conducted on these data.

## Discussion

We find no evidence for a relationship between the perceived effectiveness of health warnings and their frequency on cigarette packs. However, it is important to note that although we did not observe evidence for these practices, they may still be occurring. We were unable to obtain enough ‘female-specific’ cigarette packs in order to examine whether female-specific health warnings are found less frequently on these packs than male-specific or gender-neutral health warnings. In addition, there may be other cigarette brands, such as those which attract particular age groups, which may provide opportunities for targeted health warning placement. Similarly, we were only able to investigate if there was a relationship between health warning effectiveness and pack frequency. There may be other warning characteristics which tobacco companies may exploit to reduce the overall impact of health warnings. Finally, we did not have sufficient power to stratify our analyses by individual tobacco companies, but it is possible that if these practices occur, they may only occur at certain tobacco companies.

As far as we are aware, this is the first study to assess adherence to TPD health warning regulations using this methodology, and this proof of principle study suggests that this protocol is feasible. Although we did not find evidence for targeted placement of health warnings on cigarette packs, under both the 2001/37/EC and 2014/40/EU TPDs, these practices are technically permitted. We suggest that this potential loophole is addressed and monitoring of the placement of health warnings on cigarette packs is continued.

## References

[1] Hammond D Health warning messages on tobacco products: a review. Tob Control 2011;20:327–37. 21606180 10.1136/tc.2010.037630

[2] Noar SM Hall MG Francis DB, Pictorial cigarette pack warnings: a meta-analysis of experimental studies. Tob Control 2015, 25:341–54. 25948713 10.1136/tobaccocontrol-2014-051978 PMC4636492

[3] Doxey J Hammond D Deadly in pink: the impact of cigarette packaging among young women. Tob Control 2011, 20:353–360. 21478476 10.1136/tc.2010.038315

[4] European Commission. Pictorial Health Warnings. Available at: http://ec.europa.eu/health/tobacco/law/pictorial/index_en.htm. 15 June 2016 date last accessed.

[5] Hammond D Thrasher J Reid JL, Perceived effectiveness of pictorial health warnings among Mexican youth and adults: a population-level intervention with potential to reduce tobacco-related inequities. Cancer Causes Control 2012;23:57–67. 22362058 10.1007/s10552-012-9902-4 PMC4586036

